# Vascular service provision during the COVID-19 pandemic worsened major amputation rates in socially deprived diabetic populations

**DOI:** 10.3389/fendo.2024.1304436

**Published:** 2024-05-21

**Authors:** Ali S. AlMajali, Thomas Richards, Syed Waquar Yusuf, Bjorn Telgenkamp

**Affiliations:** ^1^ Department of Acute Internal Medicine at the Royal Free Hospital, Royal Free London NHS Foundation Trust, London, United Kingdom; ^2^ Department of Medical Education, Brighton and Sussex Medical School, Brighton, United Kingdom; ^3^ University Hospitals Sussex NHS Foundation Trust, Brighton, United Kingdom

**Keywords:** diabetic foot, social deprivation, COVID-19, amputation, surgical, public health

## Abstract

**Introduction:**

The Coronavirus Disease – 2019 (COVID-19) pandemic significantly impacted healthcare service provision and put diabetic patients at increased risk of adverse health outcomes. We aimed to assess the impact of the COVID-19 pandemic on the incidence and demographic shift of major lower-limb amputation in diabetic patients.

**Methods:**

We performed a retrospective analysis of diabetic patient records undergoing major lower-limb amputation between 01/03/2019 and 01/03/2021 at the Royal Sussex County Hospital, the regional arterial hub for Sussex. Primary outcomes were amputation incidence rates and patient demographics compared between the prepandemic and pandemic cohorts.

**Results:**

The incidence rate ratio of major lower-limb amputations shows a drop in amputations during the pandemic compared to pre-pandemic (IRR 0.82; 95% CI 0.57–1.18). Data suggests a shift in the social deprivation background of patients receiving amputations to disproportionately affect those in the more deprived 50% of the population (p=0.038). Younger patients received more amputations during the pandemic compared to prepandemic levels (p=0.001).

**Conclusion:**

Results suggest that during the COVID-19 pandemic there was a paradoxical reduction in amputations compared to prepandemic levels. However, changes to the demographic makeup of patient’s receiving amputations are alarming as younger, and more deprived patients have been disproportionately affected by the pandemic.

## Introduction

In March 2020, the World Health Organization (WHO) declared the coronavirus (COVID-19) outbreak a pandemic which triggered an immediate change in National Health Service (NHS) care processes (1). The impact of the pandemic and the associated changes on patient outcomes is largely unknown, particularly in chronic diseases where long term management and follow-up is essential. Diabetic foot care outcomes are heavily associated with service provision making them especially vulnerable to decline within the context of COVID-19 (2). Negative diabetic foot outcomes such as major lower-limb amputation contribute a significant portion of the burden of diabetes mellitus (DM) on healthcare systems (3, 4).

Our local organization, the Sussex Vascular Network (SVN) provides vascular surgery services to the population of the counties of East Sussex and West Sussex, acting as the central arterial hub for six ‘spoke’ hospitals within the region. The SVN provides vascular footcare services to a population of 1.7 million, of which 99,065 are registered diabetics with general practice surgeries (5–8). The SVN has an interest in reducing the incidence of lower-limb amputations to improve patient outcomes and reduce associated costs. Therefore, it is essential to understand the impact the COVID-19 pandemic has had on care delivery within the SVN to better guide implementation of guidelines and development of the post COVID-19 care strategy.

The primary aim was to assess the impact of the COVID-19 pandemic on DM-related major lower-limb amputations. The secondary aim was to investigate a potential relationship between the Index of Multiple Deprivation (IMD) and DM-related major lower-limb amputation in the context of COVID-19 policy changes.

## Methods

### Study design

We designed an exploratory retrospective cohort study drawing from a previous quality improvement audit and reviewed data spanning the period of 1 March 2019 to 1 March 2021. This period corresponds to the years pre- and post- the publication of “COVID-19 ‘Battle Plan’” on 1 March 2020, which was the first active public policy measure from the United Kingdom (UK) government on COVID-19 that affected NHS care provision (1). We reviewed all cases of DM-related major lower-limb amputations to assess incidence rates as compared to the national average. No sample size calculations were conducted as the data reflects the entire population of DM-related major lower limb amputations. We examined the differences in incidence rates prior to and during the period of COVID-19 policy changes to assess the impact of the pandemic on DM-related major lower-limb amputations within the SVN. We analyzed the relevant demographic data including age, sex, and deprivation to examine associations with DM-related major lower-limb amputations incidence and the impact of the COVID-19 pandemic on demographics.

### Inclusion criteria

Included cases were all major lower-limb amputations in patients with diabetic foot diseases conducted at the Royal Sussex County Hospital – the SVN major arterial hub – between 1 March 2019 and 1 March 2021. All major lower-limb amputations for diabetic foot disease for the entire region are performed at the vascular hub, as advocated in the service provision document by the Vascular Society (9). Major lower-limb amputations are defined as amputation of the lower-limb above the ankle and corresponding to codes X09.3, X09.4, and X09.5 of OPCS-4 Classification of Interventions and Procedures (10). Diabetic patients are defined as being diagnosed with type I, type II, malnutrition-related, other specified, and unspecified DM corresponding to ICD-10 codes E10-E14 (11).

Cases were obtained by requesting patient details corresponding to all operations classified as X09.3-X09.5 performed in the specified period and cross referencing against all patients with a recorded E10-E14 diagnosis. All cases of major lower-limb amputation with a recognized preceding diabetic foot complication were included. Patients with diabetic foot complications were defined as patients suffering from critical limb ischemia, diabetic foot ulcer, diabetic foot infection, diabetic foot osteomyelitis, and/or Charcot’s foot (12). Cases of bilateral amputations were included as separate cases while revisions of previous amputations were not included. Identification of the target population within these parameters was done by requesting patient details from the hospital theatre management software Bluespier and cross referencing against relevant parameters with the assistance of a data analyst.

### Exclusion criteria

Non-diabetic patients who underwent major lower-limb amputation were excluded, as were diabetic patients without coded diabetic foot disease.

### Data abstraction

A data abstraction tool was designed to capture relevant details. Sources of information included physical patient notes and electronic records. Collected data was anonymized by a unique identification number for each case. Demographic details captured included date of birth, sex, and residential postcode. Ethnicity was excluded from the demographic details captured due to inconsistent recording of ethnic origin on patient notes. Patient factors covered the relevant details of the admission including affected limb, procedure performed, date of procedure, and diabetes diagnosis.

IBM SPSS version 26 was used for processing and analysis. Age in years at the date of procedure was calculated using the date of birth, and date of procedure, and then sub-stratified into age groups: <40; 40–49; 50–59; 60–69; 70–79; and ≥80. Index of Multiple Deprivation (IMD) was captured by cross referencing residential postcodes against the English Indices of Deprivation 2019 worksheet (13). IMD was recorded by sorting data according to decile of deprivation where the 1^st^ decile represents the 10% most deprived neighborhoods in England while the 10^th^ decile represents the 10% least deprived neighborhoods.

### Data analysis

Data was dichotomized by creating pre-COVID-19 and COVID-19 cohorts, based on whether the date of procedure fell before or after 1 March 2020. Missing data were omitted from analysis. Statistical analyses were conducted using IBM SPSS version 26. Distribution of the data was determined using visual assessment of histogram distribution which showed that age was normally distributed with some left skew. Other metrics were not seen to be normally distributed. Mean (standard deviation (SD)) were reported for normally distributed data; otherwise, median (interquartile range (IQR)) was reported. The population of patients with a DM diagnosis (ICD-10 codes E10-E14) falling within the SVN catchment was calculated from General Practice surgeries registration data published in the National Diabetes Audit (14, 15). The population data was used to calculate crude incidence rates within each cohort. 95% confidence intervals (CI) for the crude incidence rates were determined using Byar’s confidence interval calculation method as recommended by Public Health England standards for the reporting of key public health measures (16). Incidence rate ratio was calculated between the two cohorts and a Wald method based approximate 95% CI was calculated (16). IMD decile data was dichotomized into deprived and non-deprived status based on falling within the 50% most deprived and 50% least deprived neighborhoods, respectively. Differences in deprivation status and sex between the cohorts were assessed using Pearson Chi-Square tests due to the data being categorical in nature. An independent samples T-Test was used to assess difference in age between the cohorts. A line graph by COVID-19 exposure was then created to map the variation of incidence over time. Bar charts were created to show variations in incidence per age group, sex, and IMD decile. **Table 1** provides a summary of all results. Data and methods were reviewed by a statistician.

**Table 1:** Summary of findings.

Metric	Pre-COVID-19	COVID-19	p Values
Incidence	63	52	N/A
Incidence Rate	6.4 per 10,000 (95% CI 4.9–8.2)	5.2 per 10,000 (95% CI 3.9–6.9)	N/A
Incidence Rate Ratio	0.82 (95% CI 0.57–1.18)		N/A
Mean Age	69 (± 11)	63 (± 9)	p=0.001
Sex	Male (55) Female (8)	Male (40) Female (12)	p=0.114
Median Deprivation	6^th^ Decile (IQR 5)	5^th^ Decile (IQR 2)	p=0.038

## Results

### Demographics

A total of 129 potential DM-related major lower-limb amputations occurring between 1 March 2019 and 1 March 2021 were found. 115 (89%) cases were included while 14 (11%) cases were excluded based on inclusion and exclusion criteria respectively. There were no missing data. 20 (17%) were female and 95 (83%) were male. All DM-related major lower-limb amputations were in adults with a mean age of 67(± 11) years (range of 33–93). Mean age within the pre-COVID-19 cohort was 69 (± 11). Mean age within the COVID-19 cohort was 63 (± 9). Median IMD lies within the 5^th^ (IQR 4) decile. There were 16 (14%) Type I diabetics and 99(86%) Type II diabetics. Of which were 6 (10%) Type I diabetics and 57 (90%) Type II diabetics within the pre-COVID-19 cohort, while there were 10 (19%) Type I diabetics and 42 (81%) Type II diabetics within the COVID-19 cohort. No other types of DM were recorded.

### Overview of incidence

**Figure 1:**
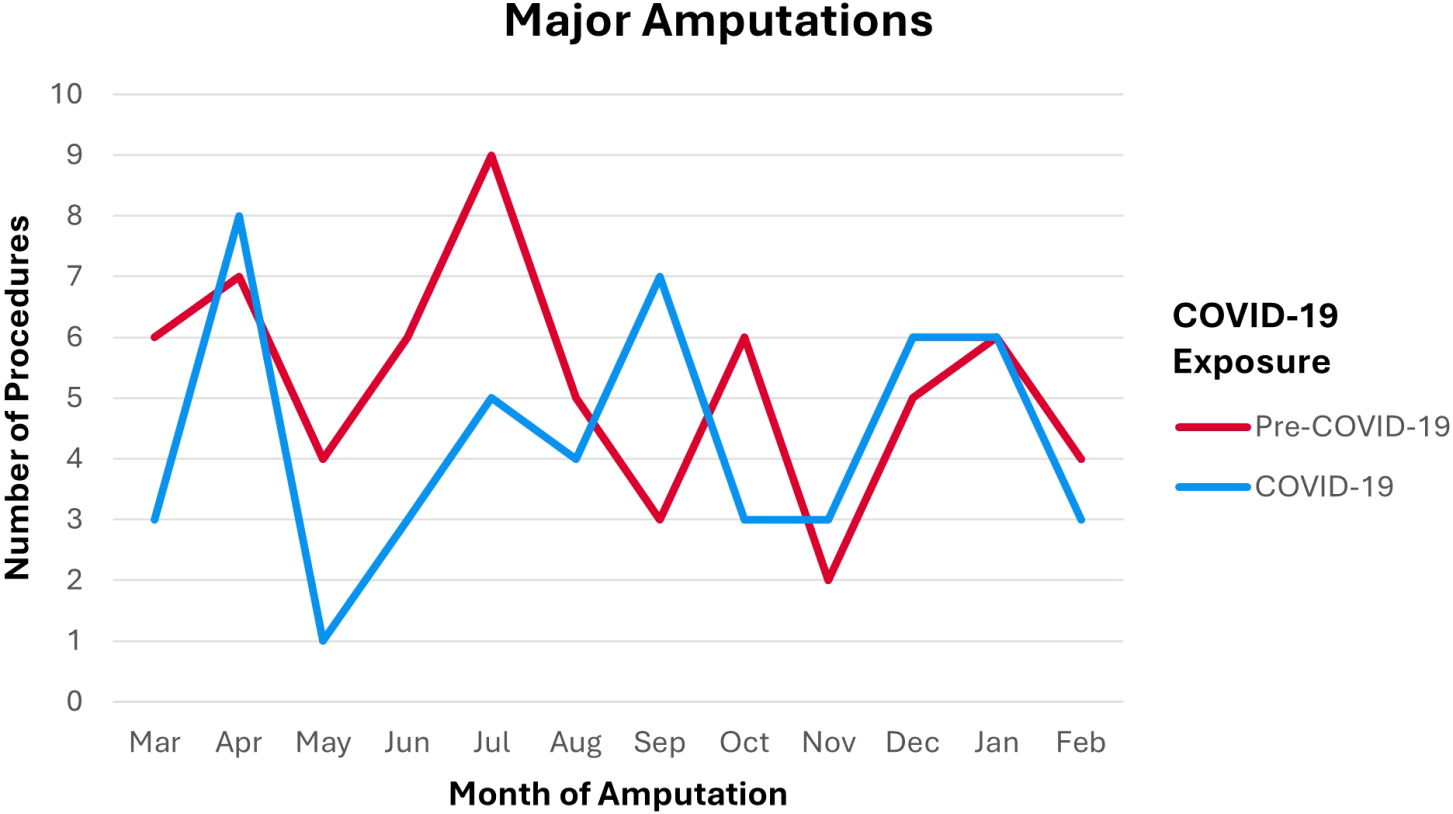
Number of DM-Related Major Lower-Limb Amputations per Month of Procedure by Cohort.

Incidence of DM-related major lower-limb amputations in the pre-COVID-19 cohort was 63. Incidence of DM-related major lower-limb amputations in the COVID-19 cohort was 52. This corresponds to an incidence rate of 6.4 per 10,000 (95% CI 4.9–8.2) pre-COVID-19 and an incidence rate of 5.2 per 10,000 (95% CI 3.9–6.9). Incidence rate ratio between pre-COVID-19 and COVID-19 cohorts was 0.82 (95% CI 0.57–1.18). Distribution of incidence over time per month of procedure by COVID-19 exposure is shown in **Figure 1**. **Figure 1** demonstrates an initial spike in number of procedures during COVID-19 in April. This elevation is followed by a large drop in May through July compared to pre-COVID-19 counts, before the number of procedures generally normalized and followed the pre-COVID-19 trends.

### Age

**Figure 2:**
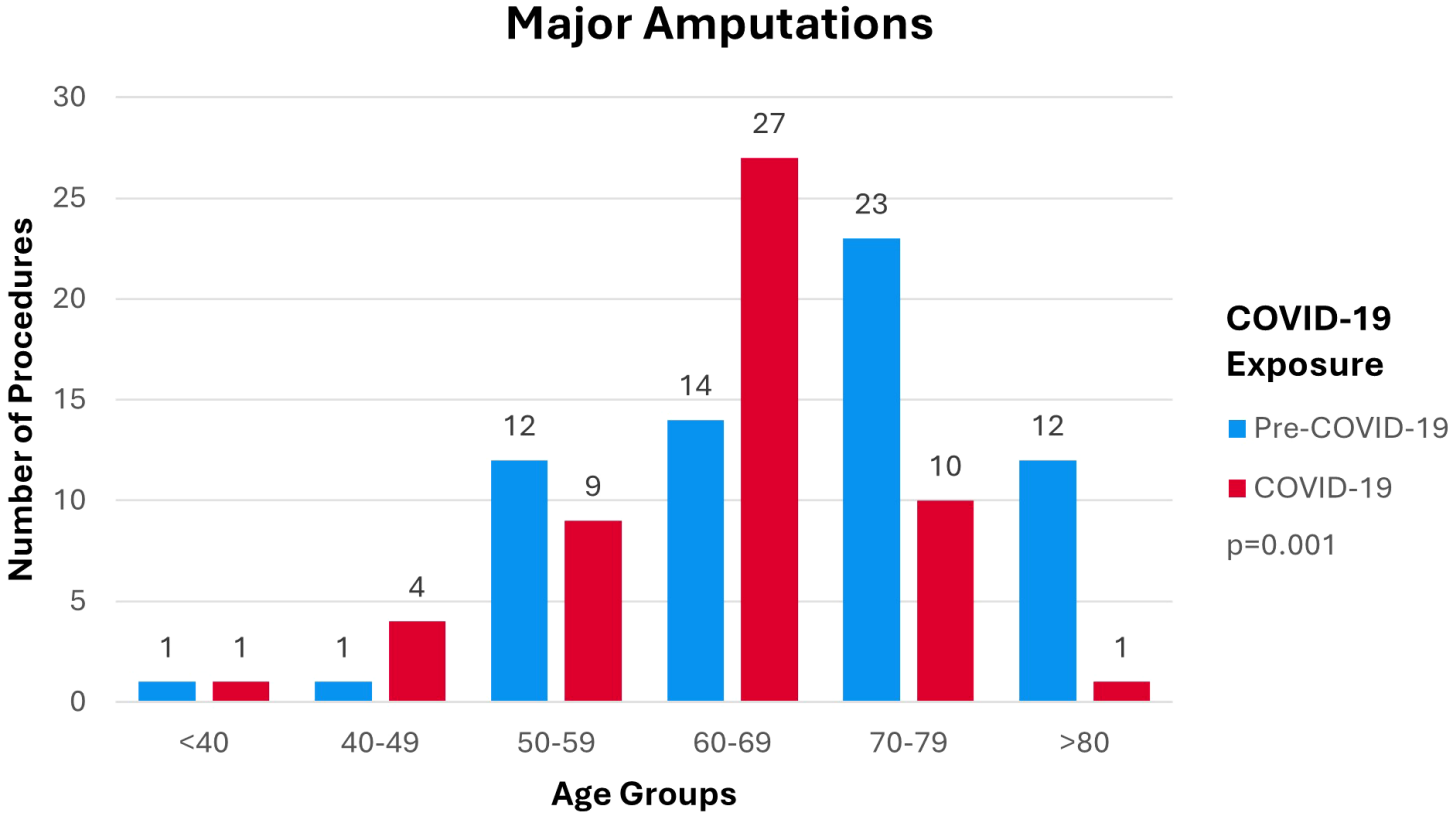
Number of DM-Related Major Lower-Limb Amputations per Age Group by Cohort.

Mean age at time of amputation within the pre-COVID-19 cohort was 69 (± 11). Mean age at time of amputation within the COVID-19 cohort was 63 (± 9). Distribution of amputation procedures per age group is shown in **Figure 2**. **Figure 2** demonstrates a shift in the distribution in the number of procedures from older age groups pre-COVID-19 to younger age groups during COVID-19 (p=0.001). Distribution of procedures performed pre-COVID-19 centers on the over 70 age groups with greater spread over all categories. Distribution of procedures performed during COVID-19 follow a more concentrated pattern centered on the 60–69 age group.

### Sex

**Figure 3:**
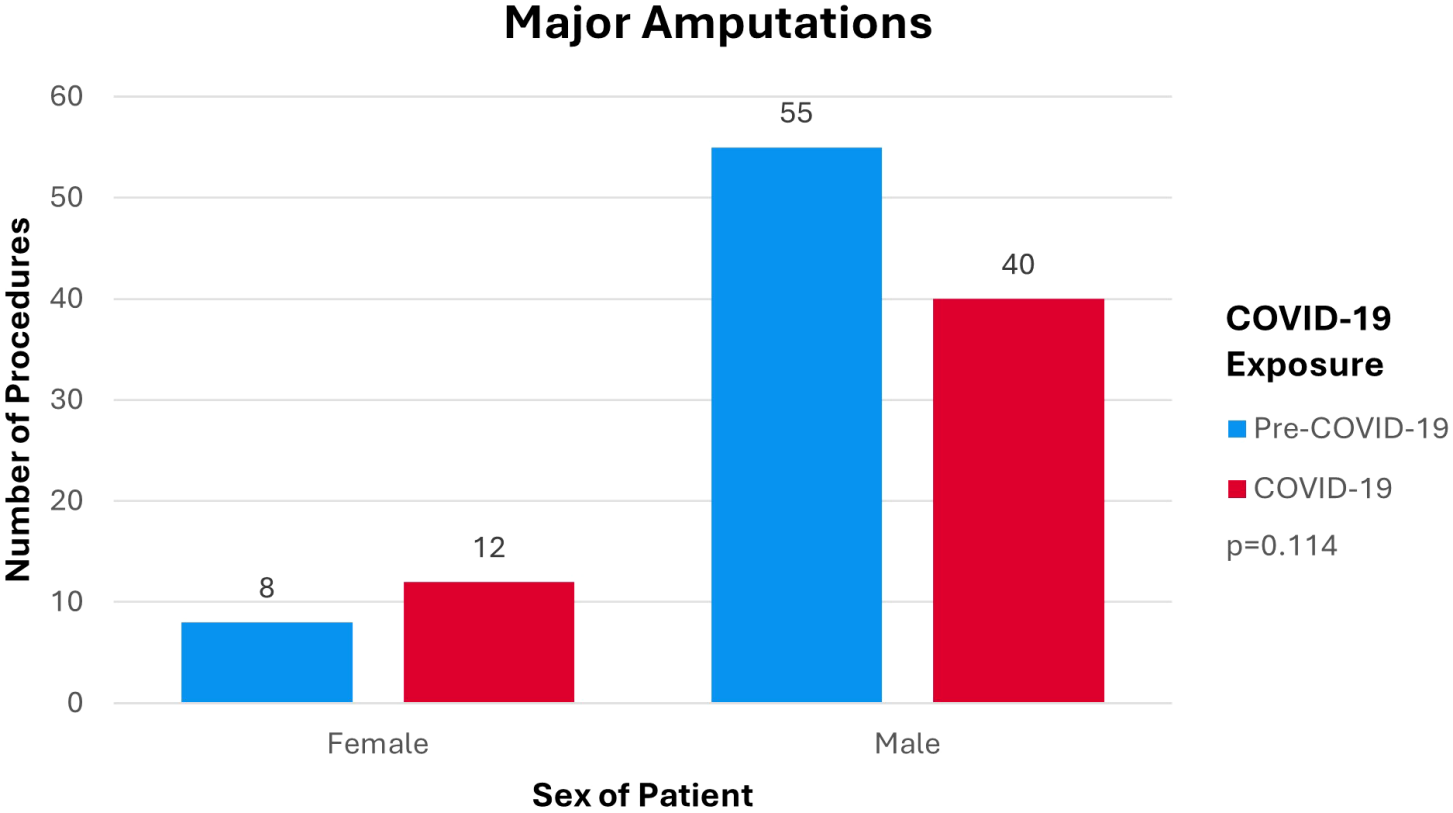
Number of DM-Related Major Lower-Limb Amputations by Sex by Cohort.

Distribution of amputation procedures by sex of patient by COVID-19 exposure is shown in **Figure 3**. **Figure 3** demonstrated no major differences in the distribution of amputations by sex between the pre-COVID-19 cohort and the COVID-19 cohort (p=0.114). The outcome of major lower-limb amputations mostly affected males as opposed to females.

### Deprivation

**Figure 4:**
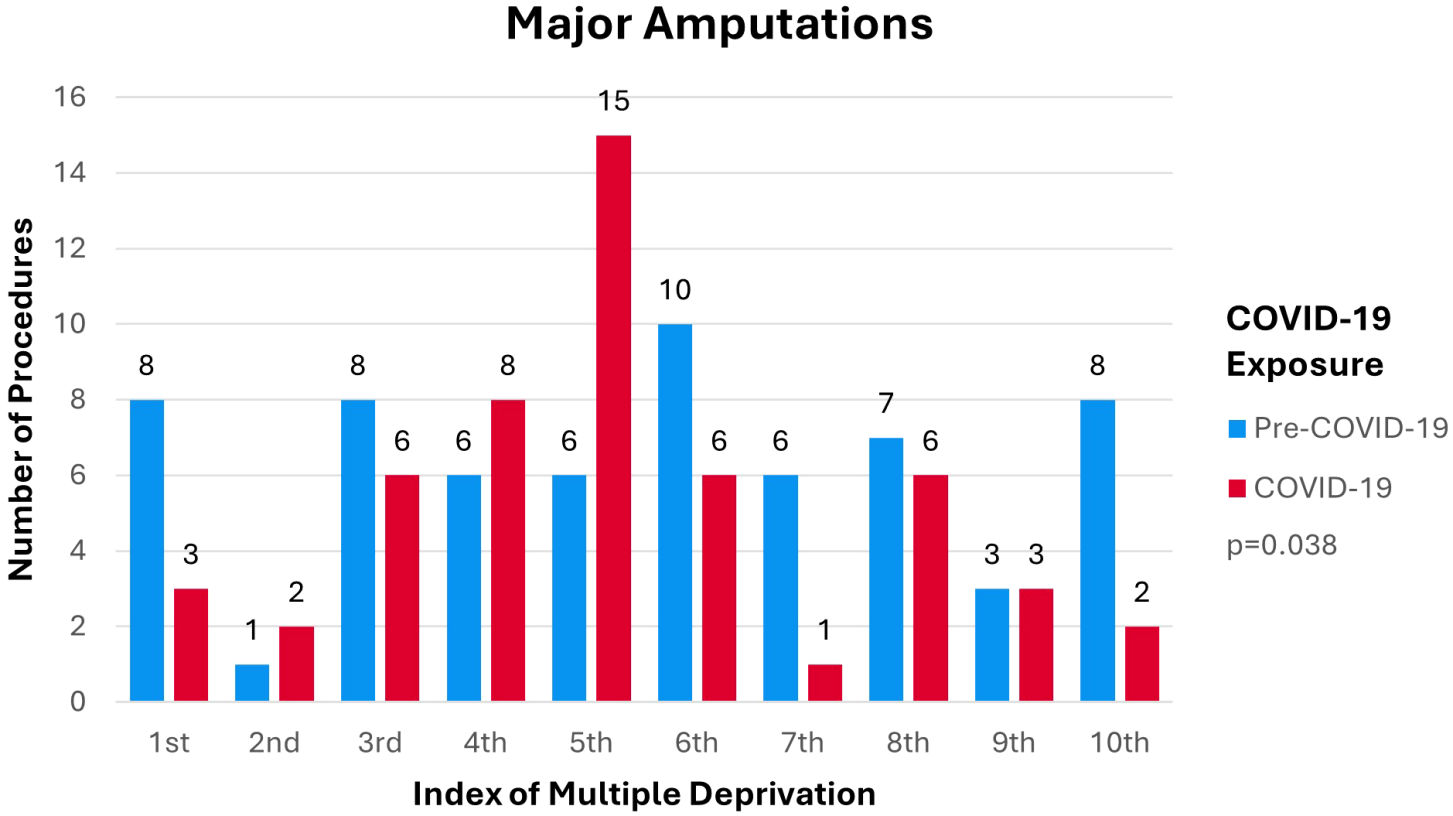
Number of DM-Related Major Lower-Limb Amputations per IMD Decile by Cohort.

Pre-COVID-19 median amputation incidence by IMD decile lies within the 6^th^ (IQR 5) decile. During COVID-19 median amputation incidence by IMD decile lies within the 5^th^ (IQR 2) decile. Distribution of amputation procedures per IMD deciles by COVID-19 exposure is shown in **Figure 4**. **Figure 4** demonstrates a shift in the number of procedures towards the 50% most deprived members of the population during COVID-19 compared to pre-COVID-19 (p=0.038). Distribution of the data across the IMD deciles pre-COVID-19 was more evenly spread amongst the different deciles. During COVID-19 the number of procedures performed became more concentrated in the 5^th^ and 4^th^ IMD deciles.

## Discussion

DM represents the 8^th^ leading cause of disability adjusted life years (DALYs) globally (17). Accounting for 2.8% of DALYs across all ages globally in 2019, it is a global public health challenge whose burden has grown by 147.9% since the 1990s (17). Within England, DM affects approximately 3.5 million individuals, costing 10% (£10 billion) of the NHS budget (4, 5, 18). Approximately 90% of the expenditure on DM patients goes to the management of associated complications such as lower limb amputations secondary to diabetic foot disease (DFD) (4). Lower-limb amputation is a preventable outcome which has a negative lifelong impact on patient quality of life and associated burden of disease on healthcare systems (19, 20). Lower-limb amputations in diabetic patients are estimated to cost the NHS £65 million spread across peri- and postoperative care excluding costs of community and prosthesis related costs (4, 18).

Many risk factors predispose patients to the development of diabetic foot ulcers and consequently to major lower-limb amputation. Clinical risk factors feature more prominently in current guidelines and literature compared to wider determinants of health (21). These wider determinants including financial insecurity, education, access to services, amongst others represent fewer collective resources within a community and are known under the umbrella of social deprivation (22). Social deprivation forms an independent risk factor for the development and subsequent prognosis of DFD (22). Literature suggests that social deprivation contributes to the development of DFD as highly as the presence of comorbid cardiovascular disease (23). Despite the high impact of social factors, they are often overlooked by clinicians and policymakers alike when approaching the question of planning diabetic care. The COVID-19 pandemic is thought to have exacerbated the role these factors play in DFD.

As the first global health emergency of the modern era, the COVID-19 pandemic has already revealed significant vulnerabilities in national healthcare systems. Global trends of diabetic lower-limb amputations increased during the pandemic which is consistent with the apparent reduced care processes seen in the NHS (24, 25). Current evidence demonstrates that some of the indirect effects of COVID-19 were seen in major reduction of primary care contacts, especially for diabetic emergencies (odds ratio 0.35) (26). Consequently, rates of health checks dropped by 76%-88% across the UK and subsequently only partially recovered (27). This manifested in reduced DM-related primary care processes including early diagnosis, monitoring, and prescribing (27). Impacts of the pandemic also included major modifications in the management approach to several vascular pathologies including DFD compared to prepandemic standards. In 4.9% of cases, these modifications lead to amputation/palliation of patients that would have been offered salvage and revascularization opportunities in prepandemic settings (28). Paradoxically, an England population wide study showed a reduction in amputation rates during the first few months following the changes associated with the pandemic (29). This is alarming given that current guidelines have not been fully implemented across the UK in the past and the extent to which this is exacerbated by COVID-19 is unknown.

The incidence of DM-related major lower-limb amputations undertaken by the SVN between 1 March 2019 to 1 March 2021 was consistently lower than the latest reported national average of 8.1 per 10,000 (7). The incidence is also lower than the previous reported statistic from the SVN of 7.2 per 10,000 (7) suggesting that diabetic footcare service provision within the SVN is better than the diabetic footcare service provision across England and is improving compared to previous years. The comparison of incidence shows a 18% drop between pre-COVID-19 and COVID-19 cohorts. **Figure 1** shows that the drop occurred within the first few months of the COVID-19 cohort before returning to previous trends. This finding is consistent with Valabhji et al’s (29) England-wide study yet inconsistent with global trends for the same period (24, 25).

The incidence of DM-related major lower-limb amputation shifted to a younger age group during COVID-19 compared to pre-COVID-19 (p=0.001). This is concerning as the COVID-19 cohort would experience more DALY’s compared to the pre-COVID-19 cohort. **Figure 3** shows incidence of DM-related lower-limb amputations predominantly in the male population compared to females in both the pre-COVID-19 and COVID-19 cohort which is consistent with the current literature (30). The dispersion of procedures shifted from a relatively equal distribution across all IMD deciles pre-COVID-19 to an increase in procedures being performed within the 50% most deprived deciles during COVID-19. This shows that during COVID-19 more deprived areas were disproportionately affected by the impact of COVID-19 on lower-limb amputation rates (p=0.038).

### Amputation incidence

Incidence of amputations from May through July during COVID-19 fell compared to the same period pre-COVID-19 levels as shown in **Figure 1**. This period contributes the most towards the decreased incidence rate ratio between the two cohorts. While this finding is consistent with the findings of Valabhji et al. (29), it is not in keeping with the available literature. Incidence was expected to increase due to decreased primary care contacts and screening of type II DM during the pandemic (26, 27). This was expected to delay diagnosis and specialist management of diabetic foot diseases resulting in more severe disease, unsalvageable limbs, and ultimately major amputations. Additionally, global trends within the same period consistently showed increased incidence of DM-related major lower-limb amputations and the UK was expected to follow the same trend (24, 25).

### Age groups and incidence

The impact of COVID-19 on DM-related major lower-limb amputations incidence within the different age groups is alarming. The shift in the dispersion of amputation incidence to a younger age group during COVID-19 as seen in **Figure 2** leads to an increase in DALYs for patients treated throughout the pandemic. This contributes negatively to the burden of DM and the burden of COVID-19 on the NHS. Those in the above 70 age groups might not have presented to services as frequently as younger age groups due to COVID-19 infection being a competing end point for morbidity and mortality in the elderly as suggested by Valabhji et al. (29). This therefore presents a confounding variable as older patients were more likely to succumb to a COVID-19 infection, and died with comorbid critical limb ischemia or diabetic foot related sepsis. Alternatively, the decreased amputation incidence might be due to those over 70 staying at home hoping to avoid COVID-19 infections at the hospital as supported by the 25.3% decreased attendance rates to emergency departments (31). The demonstrated shift in amputation incidence to age groups under 70 maybe due to a relative shift in availability of theatres for younger patients for limb saving procedures as older patients succumbed to COVID-19 or did not present to hospitals. However, absence of data to include in our analysis meant that we are unable to comment further.

### Deprivation and incidence

The correlation between IMD and DM-related major lower-limb amputations is well documented within the current literature (30). **Figure 4** demonstrates changes in the dispersion of amputation incidence towards the most deprived 50%. During COVID-19, utilization of elective admissions dropped more consistently across the different deciles while utilization of emergency admissions dropped predominantly in less deprived deciles (32). Government and healthcare responses to COVID-19, which aimed to reduce strain on services via lockdown orders and postponing elective interventions, led to an overall decrease in attendance (31, 32). Economic reports show that the COVID-19 pandemic negatively altered social determinants of health within more deprived deciles (33). These pandemic related changes may have led to increased supply of clinical resources with coinciding decreased demand for services within less deprived populations. These shifts in supply and demand characteristics are likely to have favored intervention in those falling within more deprived IMD deciles.

### Summary of COVID-19 driven trends

The COVID-19 pandemic was a novel event in modern times which allowed for real-time learning of pandemics and their effects on healthcare. Alongside the pandemic implementation of several programs such as e-consultations and telemedicine were accelerated (34). The aim of these programs was to streamline care in the context of social distancing and healthcare avoidance behaviors. The most tangible impact of COVID-19 and associated healthcare responses demonstrated by this study has been on the decreased incidence of DM-related major lower-limb amputation, especially, in the early months of the pandemic. While this may reflect that the initial efforts to decrease healthcare strain were successful, it may ultimately reveal a more negative impact on DM outcomes in the later phases of the pandemic. Despite the seemingly positive finding of decreased incidence, we have identified concerning shifts in the underlying population demographics because of COVID-19. The pandemic has disproportionately affected younger more deprived populations by altering population behaviors and healthcare provision. This is particularly pronounced as during the pandemic all-cause mortality was shown to be doubled in more deprived areas compared to equivalent populations in less deprived areas, likely as a result of the underlying health inequalities exacerbated by the onset of the pandemic (35). Whether behavioral changes will persist beyond the pandemic is yet to be seen. While the more direct impacts of COVID-19 predominantly affected morbidity and mortality in the elderly, the indirect impacts on non-COVID-19 related pathology are unclear.

### Equity and outcomes

As a wider determinant of health, deprivation plays a key role in forecasting patient outcomes at the population level. It is important to acknowledge that deprivation extends beyond low income, to encompass a lack of socioeconomic resources and adverse environmental circumstances to good living. The latest data from the Office for National Statistics suggests that differences in healthy life expectancy at birth between individuals living in the least and most deprived areas amounts to approximately two decades (36). The statistics also show that while overall life expectancy at birth is increasing, the inequality gap between individuals living in the least deprived and most deprived areas is widening as well (36). This is especially alarming for patients suffering from diabetic foot problems as current literature indicates that individuals living in the most deprived areas are at higher risk of receiving a major lower-limb amputation compared to those in the least deprived areas (37). Despite this information, the impact of deprivation is compounded by lower service access and utilization inequities even in countries with established universal healthcare (38).

The current state of diabetic foot care necessitates a renewed and more equitable approach that can adapt to the ever-changing characteristics of the population. Given the rise in amputations amongst the younger and more deprived during the pandemic, this study suggests that current pathways failed to adapt to shifts in healthcare demand. The future brings increasing population demands and patient complexity which requires dynamic care pathways that can respond to the challenges of the time. The current NICE diabetic foot guidelines establish the foundational structures, and processes for services and treatment thresholds for patients while leaving implementation details to the individual partnerships (21). Therefore, it falls upon the partnerships to generate services that go beyond the clinical details and can stratify patients according to wider determinants of health in pursuit of optimal and equitable outcomes for all.

### Strengths and limitations

This study sat within a wider desire to expand the understanding of footcare service provision within the SVN and the unique demographic characteristics of the target population in the context of COVID-19. Due to the fact all relevant operations were undertaken at the Royal Sussex County Hospital, it was possible to include all cases of DM-related major lower-limb amputations within the specified period. This eliminates any associated sampling bias thereby improving the internal validity of the results. Findings are likely to be representative of trends within the wider population of England and the UK at large as the data captures all DM-related major lower limb amputations from a population of 1.7 million (8). As the data captures the entire population of patients receiving major lower limb amputation secondary to DFD no sample size calculation was completed. This opens potential for type 2 errors to be present within the findings. This limits the generalizability of the findings to the wider population as a whole. However, given the congruence of findings from this study and others exploring pandemic related outcomes within the same respective timeframe. The findings are limited to the trends occurring within a year of the COVID-19 pandemic starting. The impact of the COVID-19 pandemic on DM-related major lower-limb amputations would likely extend beyond this period potentially leading to short-sighted findings, however, it is important to note that this study is among the few that investigate the effects of COVID-19 on a specific healthcare outcome alongside underlying trends in demographic data.

### Implications

The changes in amputation incidence across age groups and IMD deciles prompts further investigation to develop an understanding of the underlying causes. This should ideally include ethnicity as a confounder to explore its impact. It is also important to explore the impact of the changes to healthcare provision that may have been beneficial to guide future provision. Variation in trends over time also warrants further research of a future period to assess the external validity of this study’s findings over time and the impacts of COVID-19 deeper into the pandemic period.

## Conclusion

We have demonstrated decreased incidence rates of DM-related major lower-limb amputations within the SVN prior to and during the COVID-19 pandemic compared to previous local and national statistics. COVID-19 driven trends in age group and deprivation characteristics of the reference population highlight the impact of the pandemic on DM footcare service provision. Demographic changes were notably concerning and warrant further investigation into the longer-term impacts of COVID-19. The finding of inequal outcomes between groups of varying deprivation requires specific investigation to ensure equitable provision of services.

## Data availability statement

The original contributions presented in the study are included in the article/supplementary material. Further inquiries can be directed to the corresponding author.

## Ethics statement

Ethical approval was not required for the study involving humans in accordance with the local legislation and institutional requirements. Written informed consent to participate in this study was not required from the participants or the participants’ legal guardians/next of kin in accordance with the national legislation and the institutional requirements.

## Author contributions

AA: Writing – original draft, Writing – review & editing, Investigation, Resources, Software, Visualization. TR: Writing – review & editing, Data curation, Formal analysis, Supervision. SY: Supervision, Writing – review & editing, Conceptualization. BT: Conceptualization, Funding acquisition, Methodology, Supervision, Writing – review & editing.
